# FP-YOLOv8: Surface Defect Detection Algorithm for Brake Pipe Ends Based on Improved YOLOv8n

**DOI:** 10.3390/s24248220

**Published:** 2024-12-23

**Authors:** Ke Rao, Fengxia Zhao, Tianyu Shi

**Affiliations:** School of Mechanical and Power Engineering, Zhengzhou University, Zhengzhou 450000, China; rk15516978289@gs.zzu.edu.cn (K.R.); sty@gs.zzu.edu.cn (T.S.)

**Keywords:** brake pipe ends, surface defect detection, YOLOv8n, label assignment

## Abstract

To address the limitations of existing deep learning-based algorithms in detecting surface defects on brake pipe ends, a novel lightweight detection algorithm, FP-YOLOv8, is proposed. This algorithm is developed based on the YOLOv8n framework with the aim of improving accuracy and model lightweight design. First, the C2f_GhostV2 module has been designed to replace the original C2f module. It reduces the model’s parameter count through its unique design. It achieves improved feature representation by adopting specific technique within its structure. Additionally, it incorporates the decoupled fully connected (DFC) attention mechanism, which minimizes information loss during long-range feature transmission by separately capturing pixel information along horizontal and vertical axes via convolution. Second, the Dynamic ATSS label allocation strategy is applied, which dynamically adjusts label assignments by integrating Anchor IoUs and predicted IoUs, effectively reducing the misclassification of high-quality prediction samples as negative samples. Thus, it improves the detection accuracy of the model. Lastly, an asymmetric small-target detection head, FADH, is proposed to utilize depth-separable convolution to accomplish classification and regression tasks, enabling more precise capture of detailed information across scales and improving the detection of small-target defects. The experimental results show that FP-YOLOv8 achieves a mAP50 of 89.5% and an F1-score of 87% on the ends surface defects dataset, representing improvements of 3.3% and 6.0%, respectively, over the YOLOv8n algorithm, Meanwhile, it reduces model parameters and computational costs by 14.3% and 21.0%. Additionally, compared to the baseline model, the AP50 values for cracks, scratches, and flash defects rise by 5.5%, 5.6%, and 2.3%, respectively. These results validate the efficacy of FP-YOLOv8 in enhancing defect detection accuracy, reducing missed detection rates, and decreasing model parameter counts and computational demands, thus meeting the requirements of online defect detection for brake pipe ends surfaces.

## 1. Introduction

Currently, most automotive braking systems employ hydraulic brakes, with brake lines primarily connected and sealed through pipe nozzles at the brake pipe ends. However, during manufacturing, defects such as cracks, scratches, flashes, or incomplete skin removal may appear on the pipe ends’ surface. Such defects can lead to air or fluid leakage, resulting in insufficient brake pressure, reduced braking performance, and ultimately impacting the safe operation of the braking system while increasing maintenance costs. Thus, detecting end surface defects is essential during the manufacturing process. Nevertheless, the complexity and diversity of these defect features, with varying backgrounds and colors, as well as the generally small size of cracks and scratches, create significant challenges for defect detection. Additionally, to meet practical production requirements, the inspection system must achieve high accuracy and complete the inspection within a short timeframe, further complicating the detection process.

Detection of surface defects on ends is one of the visual inspection tasks. Early detection algorithms distinguish defects from background regions by analyzing image features such as texture, edges, shape, color, and spectrum [1]. Although this approach has a low computational demand, selecting the optimal threshold relies on manual inputs, thus increasing the subjectivity and uncertainty of the detection results. Moreover, when the target or environment changes, traditional detection methods often require reconfiguration or redesign, and shallow feature-based approaches are highly susceptible to noise or uneven illumination, which can blur or fragment target boundaries [2]. To enhance adaptability, researchers have applied machine learning methods inspired by the learning mechanisms of the human brain, enabling computers to learn from data and recognize a variety of defect types, even with novel data. Traditional machine learning models, such as HAAR [3], HOG [4], DPM [5], SURF [6], and SIFT [7], have been widely used in object detection, where intricate feature extraction and algorithmic design allow for high accuracy. However, these models often struggle in complex environments with significant variations. For instance, the HAAR-based cascade classifier is sensitive to illumination changes and occlusion, leading to a significant drop in detection accuracy. In a study by Viola and Jones [8], the HAAR detector performed well under controlled lighting conditions, but exhibited lower precision when tested on images with dynamic lighting or objects in motion. Similarly, HOG features, which are effective for object detection in static images, fail to deliver robust performance when objects are occluded or deformed, as shown in the work by Dalal and Triggs [9], where detection accuracy decreased by over 30% when objects were partially obscured. Additionally, these traditional models require manual feature selection and intensive parameter tuning, which limits their scalability to large, diverse datasets.

With rapid advancements in deep learning in computer vision, convolutional neural networks (CNNs) have demonstrated exceptional capabilities in classifying, detecting, and segmenting industrial defects. Efficient neural network design is critical for accurately recognizing end surface defects. CNN-based detection techniques effectively capture both global and local information by extracting and integrating multiscale features, significantly enhancing the model’s representational power. Through successive convolution and pooling operations, CNNs can identify key characteristics such as object size, shape, and texture, enabling precise localization and classification of defects during manufacturing processes. Consequently, CNN-based methods have been extensively applied and studied in industrial surface defect detection. Notably, the YOLO [10] series of models has gained significant attention for its efficiency and accuracy, achieving a favorable balance between real-time performance and precision, making it a robust solution for defect detection in complex industrial environments. For example, Lu et al. [11] introduced an enhanced YOLOv5 algorithm tailored for detecting small steel surface defects. They included the RepVGG (Re-param VGG) module to improve model robustness and expressiveness; moreover, they replaced the backbone network with FasterNet to achieve high inference accuracy and speed for real-time monitoring. A genetic algorithm (GA) was paired with an OTA loss function and model pruning to reduce model size while improving generalization and defect feature capture. Similarly, Chu et al. [12] employed YOLOv8 as a foundation for steel surface defect detection, substituting the original backbone with StarNet for lightweight optimization without sacrificing accuracy. They also added an occlusion-aware attention mechanism, SEAM, to the detection head, enabling the model to handle occluded object features more effectively and perform well in complex environments. Tie et al. [13] proposed LSKA_YOLOv8, a lightweight steel defect detection model incorporating KWConv and a large separable kernel attention (LSKAttention) module in the detection head, which enhanced target feature comprehension and boosted model performance in defect identification.

Beyond the application in defect detection, YOLO-based models have been widely adopted in various fields due to their real-time detection capabilities and robustness. For instance, Chen et al. [14] applied YOLOv8 to the RailFOD23 dataset for foreign object detection on railroad transmission lines, achieving excellent performance. He et al. [15] proposed ALSS-YOLO, a lightweight detector for TIR UAV applications, incorporating ALSS, LCA, and a single-channel focus module to enhance feature extraction for blurry and overlapping small targets. Extensive experiments on the BIRDSAI and ISOD UAV wildlife datasets showed its state-of-the-art performance.

Despite the existing developments in the field, the detection of end surface defects continues to pose significant challenges. These challenges stem from several factors. The small size of the targets makes them easily overlooked, while their unique characteristics require specialized detection methods. The variable positions of the defects within the image add complexity to the detection process. Moreover, cluttered backgrounds often interfere with the accurate identification of the defects, and strict time constraints limit the available computational resources and processing time. Consequently, the current CNN-based detection methods for end surface defects struggle to achieve satisfactory results. To overcome these obstacles, this paper proposes a novel object detection model, FP-YOLOv8. The main contributions of this paper are as follows:

1. To meet the demands of lightweight design, we suggest replacing the C2f module in the YOLOv8 model with C2f_GhostV2. This modification results in fewer model parameters, reduced computational load, and enhanced feature representation capabilities.
2. The introduction of the Dynamic ATSS label allocation strategy is another key in-novation. This strategy dynamically adjusts label assignment, enhancing detection accuracy and reducing the misdiagnosis rate.
3. A new detection head, FADH, is presented in this paper to increase the accuracy of detecting small objects in complex backgrounds by capturing comprehensive information at various sizes.

## 2. Related Work

### 2.1. Object Detection

In recent years, rapid advances in deep learning have driven significant success in a variety of computer vision applications, including picture classification, semantic segmentation, instance segmentation, and object recognition. Among these, object recognition remains a particularly difficult task in computer vision, since it needs to detect item types inside images and precisely localize these things. Object detection algorithms are categorized into single-stage and two-stage types, depending on their structural design and underlying concepts. Single-stage detectors execute object classification and bounding box regression directly on retrieved feature maps, resulting in faster processing and simpler architecture. Representative single-stage models include YOLO, SSD [16], and Retina-Net [17]. In contrast, two-stage detectors perform object identification in two steps: candidate region generation and fine-grained categorization. Potential bounding boxes are created during candidate region extraction and then classed and localized for accuracy. Notable two-stage models include SPPNet [18], Faster-RCNN [19], Mask-RCNN [20], and R-FCN [21]. While two-stage detectors tend to achieve higher detection accuracy, their complex structures and longer inference times hinder practical applications. Generally, CNN-based detection algorithms rely on feature extractors such as VGG [22] and ResNet [23] as a backbone [24]. These methods increase network depth, integrate multiscale feature for fusion, and introduce additional detection branches to enhance model performance. Despite their robust performance on public datasets, these algorithms encounter challenges in detecting complex industrial surface defects, which necessitates further optimization of the network structure to better accommodate industrial defect characteristics.

Although single-stage defect detection models are advantageous in terms of rapid detection, their accuracy still has room for improvement. By optimizing the network structure, we can enhance the defect detection accuracy while maintaining the model’s efficiency. Thus, the goal of this work is to meet the high-accuracy and high-efficiency requirements for defect identification in this industry by improving the YOLOv8 model’s detection accuracy, and also meet the lightweight requirement for end surface defects.

### 2.2. Defect Detection

With rapid developments in industrial automation and intelligent technology, deep learning-based defect detection has gained extensive application across industrial fields. For instance, Dong et al. [25] proposed a digital twin-assisted multiscale residual-self-attention feature fusion network (MRFFN) for hypersonic flight vehicle (HFV) fault diagnosis. They constructed a digital twin model to simulate HFV fault conditions and generate realistic training data. To improve fault diagnosis performance, they introduced multiscale structures and GRU into the convolutional neural network and designed a residual-self-attention mechanism to focus on key features. Wang et al. [26] proposed a dynamic collaborative adversarial domain adaptation network (DCADAN) for unsupervised fault diagnosis in rotating machinery. The model features a dynamic generator for adaptive feature extraction, a dual-system adversarial framework for task-specific adjustments, and a multi-source domain loss for efficient cross-domain diagnosis. Dong et al. [27] developed an interpretable multiscale lifting wavelet contrast network for planetary gearbox fault diagnosis. The model uses a lifting wavelet layer for feature decomposition, an interactive channel attention mechanism to select frequency-specific features, and a time–frequency contrast loss to improve feature distribution. Wang et al. [28] proposed a trackable multi-domain collaborative generative adversarial network (TMCGAN) for fault diagnosis in rotating machinery with imbalanced data. TMCGAN enhances interpretability and credibility through a multi-domain adversarial strategy for comprehensive feature learning, parallel frequency loss for enriched training feedback, and a streaming tracking factor for real-time decision rationale. Ma et al. [29] proposed a method for detecting complex flaw clusters using a three-step analysis with dynamic XFEM. The approach combines the IDABC-HCA algorithm for flaw detection, BFGS for geometry refinement, and local mesh refinement to improve accuracy and reduce computational cost, demonstrating efficiency in flaw detection. Wang et al. [30] proposed the GhostConvML (GCML) module to replace conventional convolution blocks in YOLOv8, thus improving the model’s generalization in feature extraction. They also designed the Alpha-EIoUs loss function, which accelerates model convergence and enhances the accuracy of casting surface defect identification. Moreover, Lang et al. [31] presented a lightweight YOLOv5 variant (MR-YOLO). They incorporated Mobilenetv3 in the neck to reduce FLOP and enhance feature expression. Mosaic data augmentation was used and the SPPF was replaced with the SE module to improve robustness and better capture small defects.

Based on the structural modification and performance-enhancing strategies in the previous research, the C2f_GhostV2 module was constructed in this paper to replace the C2f module in YOLOv8. Additionally, a new FADH detection head was proposed to further improve the detection performance of the surface defects on the ends.

### 2.3. Label Assignment

Label assignment is a decisive factor influencing the performance of object recognition models, as the classification of positive and negative samples directly impacts network learning and convergence. Models such as Faster R-CNN, SSD, and Retina-Net typically use a fixed threshold for IoUs to assign labels. For instance, during the training of Faster R-CNN, anchors having an IoU greater than 0.7 with any ground-truth bounding box are labeled as positives, those with an IoU less than 0.3 are regarded as negatives, and anchors with IoUs falling between 0.3 and 0.7 are disregarded [32]. While straightforward, this fixed-threshold approach overlooks variations in object shape, size, and the number of corresponding positive anchors. For example, objects with regular shapes or larger sizes yield more high-quality positive anchors and thus receive more attention during training. In contrast, slender or small objects often lack sufficient high-quality anchors, causing the network to focus on detecting objects with balanced aspect ratios or larger dimensions, which limits performance on slender or small objects.

Recently, researchers have focused on developing adaptable thresholds and gradually removing fixed thresholds for label assignments. ATSS [33] determines adaptive thresholds by utilizing the mean and standard deviation of the IoUs distribution between candidate anchors and ground-truth objects. In contrast, PAA [34] evaluates anchor boxes by combining classification and localization scores. PAA delivers accurate positive–negative sample classification by fitting high-score candidate boxes to a Gaussian Mixture Model (GMM) and improving it using the Expectation-Maximization technique. While adaptive threshold approaches improve detection performance, prediction-based label assignment holds the potential for increased accuracy.

Dynamic ATSS was introduced in this paper. By introducing the IoUs of prediction boxes to dynamically adjust the division criteria of positive and negative samples, the model can better adapt to different object characteristics and scenarios. The adaptability and accuracy of label assignments were further improved.

## 3. Methods

### 3.1. C2f_GhostV2

**Figure 1:**
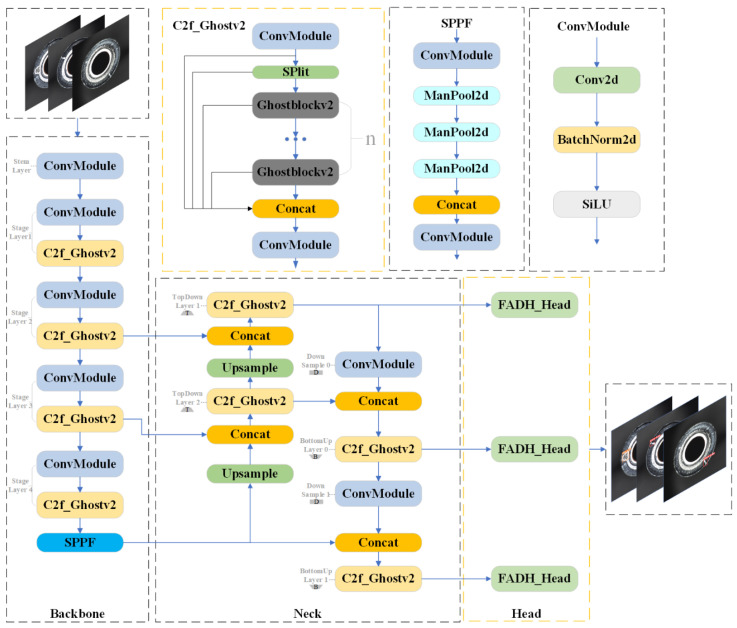
The structure of FP-YOLOv8.

YOLOv8 employs traditional convolution and C2f modules to extract and down-sample high-quality image features. However, the up-sampling process within the neck, combined with the utilization of Bi-PAN-FPN [35], increases both the parameter count and the overall complexity of the model. To create a more lightweight YOLOv8n detection network, improve processed speed, and ensure performance on small-target detection, the YOLOv8n algorithm is enhanced in this paper, and the FP-YOLOv8 model is proposed, as shown in Figure 1.

**Figure 2:**
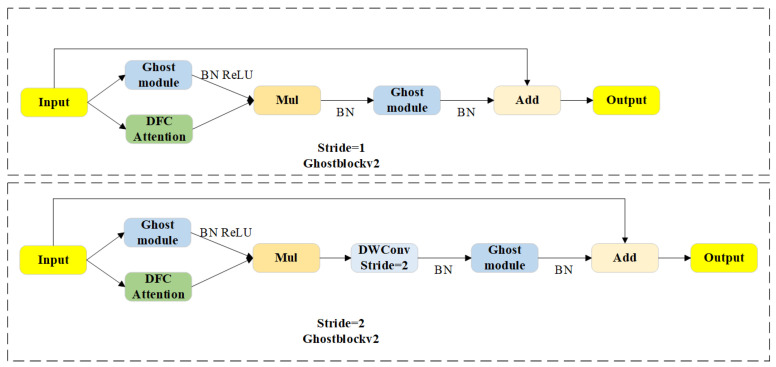
The structure of the Ghostblockv2.

As shown in Figure 1, in the FP-YOLOv8 model, the C2f module in the original model is replaced with C2f_GhostV2, which is constructed based on the lightweight model Ghost-NetV2 [36]. Specifically, the C2f_GhostV2 module optimizes the network by substituting the bottleneck component in C2f with the Ghostblockv2 module (as shown in Figure 2). The core of Ghostblockv2 is to reduce redundant calculations through the Ghost module and enhance the feature expression ability in combination with the DFC attention mechanism, especially in capturing long-distance dependent information. Compared with the traditional C2f module, C2f_GhostV2 improves the network’s lightweight performance and maintains detection accuracy.

**Figure 3:**
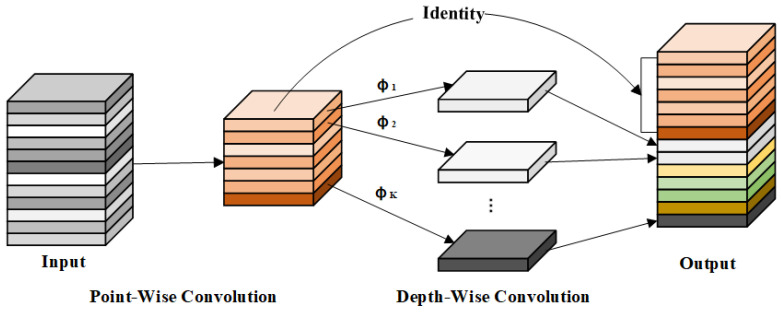
The principles of the cheap operations. Cheap operations use point convolution and depth convolution to obtain more feature maps with less computational cost.

**Figure 4:**
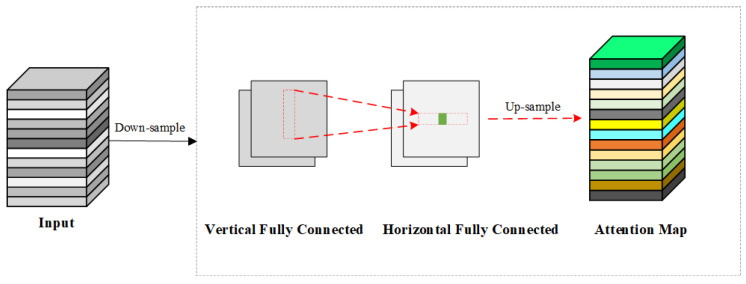
The principles of the DFC attention mechanism. The horizontal and vertical Fully Connected layers capture the long-range information along the two directions, respectively.

Ghostblockv2 employs the Ghost module for cheap operations, and its implementation process is presented in Formulas (1) and (2):

(1) $$Y\prime =X*F_{1\times 1}$$

(2) $$Y=Concat(Y\prime ,Y\prime *F_{dp})$$

where $F_{1\times 1}$ represents point-wise convolution; $F_{\mathrm{dp}}$ represents depth-wise convolution; $X\in {\mathbb{R}}^{H\times W\times C}$ is the input feature; $Y\prime \in {\mathbb{R}}^{H\times W\times C_{out}^{\prime }}$ are the intrinsic features; $Y\in {\mathbb{R}}^{H\times W\times C_{out}}$ is the output feature; and $C_{out}^{\prime }<C_{out}$. In contrast to traditional convolution, the Ghost module combines point-wise and depth-wise convolutions to reuse features and reduce computational costs, as shown in Figure 3, although it limits spatial information expression. To overcome this limitation, the DFC attention mechanism is incorporated to improve the model’s ability to capture spatial dependencies within the features. The detailed process is described in Formulas (3) and (4).

(3) $$a\prime_{hw}=\sum_{h\prime =1}^{H}F_{h,h\prime w}^{H}⊙X_{h\prime w},h=1,2,\cdot \cdot \cdot ,H,w=1,2,\cdot \cdot \cdot ,W$$

(4) $$a_{hw}=\sum_{w\prime =1}^{W}F_{w,hw^{\prime }}^{W}⊙a\prime_{hw\prime },h=1,2,\cdot \cdot \cdot ,H,w=1,2,\cdot \cdot \cdot ,W$$

where $X\in {\mathbb{R}}^{H\times W\times C}$, it is consistent with the input in Formula (1). $F^{H}$ and $F^{W}$ are the transformation weights in the horizontal and vertical directions, respectively. The original features are first processed through Equations (3) and (4) successively to capture long-range dependencies along two distinct directions. This method, known as decoupled fully connected (DFC) attention, is illustrated in Figure 4. DFC attention captures pixel information along horizontal and vertical axes separately, efficiently implemented via convolution to reduce computational complexity and establish indirect relationships among patches within a square region, thus improving inference speed compared to global attention.

Finally, the attention map generated by DFC attention is normalized to (0,1) by Sigmoid and multiplied element-wise with the feature map V(X) generated by the cheap operation to obtain the final output:

(5) O=Sigmoid(A)⊙V(X)

Ghostblockv2 combines cheap operations with DFC, reducing the model’s complexity while taking into account the global information of features.

### 3.2. Dynamic ATSS

Owing to the complexity and diversity of ends surface defects, which are characterized by a complicated background, irregular shapes, and varying sizes, detecting these defects presents significant challenges. As the same defect may exhibit varying characteristics, correct label assignment becomes crucial for effective detection. To address this issue, we introduce Dynamic ATSS [37] (Dynamic Adaptive Training Sample Selection), an enhanced label assignment strategy designed to improve the performance of object detection models. The traditional ATSS method calculates adaptive thresholds based solely on the IoUs’ distributions between candidate anchors and ground-truth objects to determine positive and negative samples. However, this approach overlooks the quality of the predicted boxes, potentially leading to high-quality predicted samples being misclassified as negative samples. To overcome this limitation, Dynamic ATSS incorporates Predicted IoUs to dynamically adjust the classification criteria for positive and negative samples, enhancing label assignment accuracy.

The core concept behind Dynamic ATSS is the integration of Anchor IoUs and Predicted IoUs to determine final label assignments. Specifically, it calculates the Combined IoUs using the following formula:

(6) CIoUs=PIoUs+AIoUs

where PIoUs signifies the IoU of predicted bounding boxes to ground-truth boxes, and AIoUs denotes the IoU of pre-defined anchor boxes to ground true boxes. In order to calculate the adaptive label assignment thresholds, Dynamic ATSS performs the calculates of mean and standard deviation for PIoUs and AIoUs, respectively:

(7) mean(CIoUs)=mean(PIoUs)+mean(AIoUs)

(8) std(CIoUs)=std(PIoUs)+std(AIoUs)

(9) threshold(CIoUs)=mean(CIoUs)+std(CIoUs)

**Figure 5:**
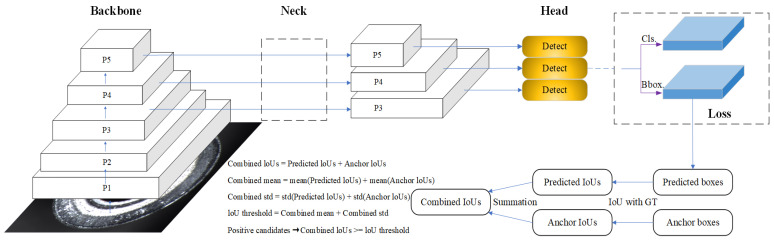
Dynamic ATSS network architecture diagram. Dynamic ATSS uses the predicted boxes decoded from the regression branch. The predicted IoUs and anchor IoUs are calculated by comparing the predicted and anchor boxes with the GTs. The Combined IoUs (CIoUs) are obtained by summing the predicted and anchor IoUs. The combined mean and std are calculated similarly. The IoU threshold is the sum of the combined mean and std, and positive candidates are defined as samples with Combined IoUs greater than or equal to the threshold, restricted within the ground truth bounding boxes as final positive samples.

Once the threshold for the combined IoUs is determined, any sample with an IoU that meets or exceeds this threshold is categorized as a positive sample. The network architecture of Dynamic ATSS is shown in Figure 5. The method extracts regression results, decodes the regression offsets into the coordinates of the predicted box, and computes the IoUs between the predicted bounding boxes and the ground truth boxes, denoted as CIoUs.

Dynamic ATSS is remarkable due to its ability to adaptively integrate Anchor IoUs with Predicted IoUs, which helps in selecting high-quality positive samples during training. In the early phase of training, due to random initialization, predictions are often inaccurate, and anchors offer essential guidance. As training progresses, the influence of predictions gradually grows, thus playing a dominant role in the Combined IoUs. This adaptive mechanism reduces the likelihood of misclassifying high-quality predicted samples as negative, thus enhancing the overall performance of the detection model.

### 3.3. Fine-Grained Asymmetric Detection Head (FADH)

**Figure 6:**
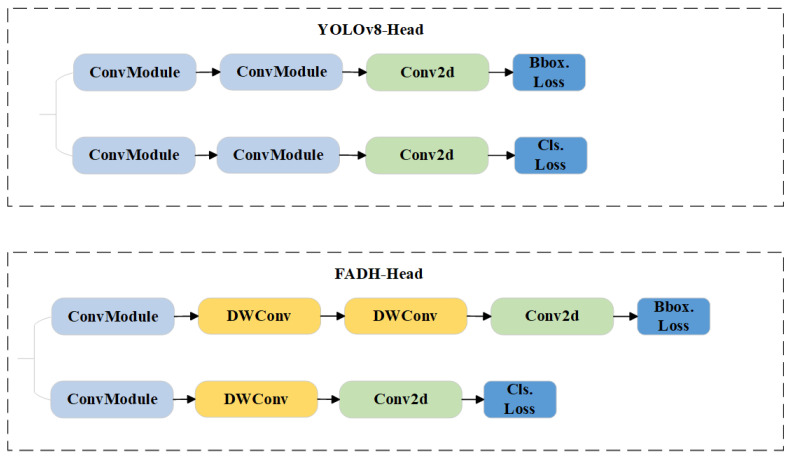
The structure of the YOLOv8 detection head and FADH detection head.

Since most surface defects on brake pipe ends, particularly cracks and scratches, are small (typically around 0.02 mm), they present a significant challenge for defect detection. To address this issue, an efficient and lightweight small-target detection head, FADH (Fine-grained Asymmetric Detection Head), is proposed in this paper to enhance small-target detection performance while maintaining model efficiency and a lightweight design. FADH introduces 3 × 3 depth-wise separable convolution (DWConv) [38] to replace part of the traditional 3 × 3 standard convolution. DWConv’s strength lies in breaking down the convolutional process into depth-wise and pointwise convolutions, which facilitates separate feature extraction in the spatial dimension and feature fusion in the channel dimension. In contrast to standard convolution, FADH enhances the model’s capacity to capture fine-grained information at several scales while lowering the computational load and parameter count. This optimization allows for the model to maintain high performance with less computational expense. As a result, FADH not only streamlines feature extraction, but also boosts the accuracy of small-object detection. The FADH structure is illustrated in Figure 6.

FADH employs a decoupled design for the detection head. It separates the classification task and the bounding box regression task into distinct network branches, which are learned separately and later fused. Combined with depth-wise separable convolutional layers, this design prevents the over-expansion of the two tasks and alleviates task coupling. By reducing the coupling between tasks, the correlation loss for positive samples becomes more targeted, thereby improving the detection accuracy of smaller targets. After replacing the detection head in YOLOv8 with FADH, the network enhances small-target defect detection accuracy while maintaining efficient detection performance.

## 4. Experimental Result and Discussion

### 4.1. Dataset

**Figure 7:**
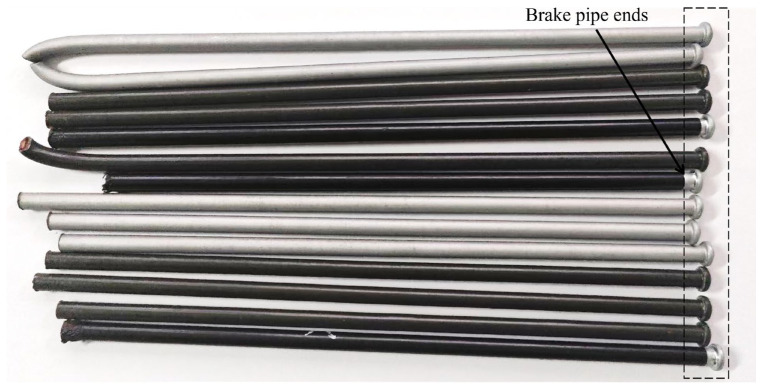
Brake pipes.

**Figure 8:**
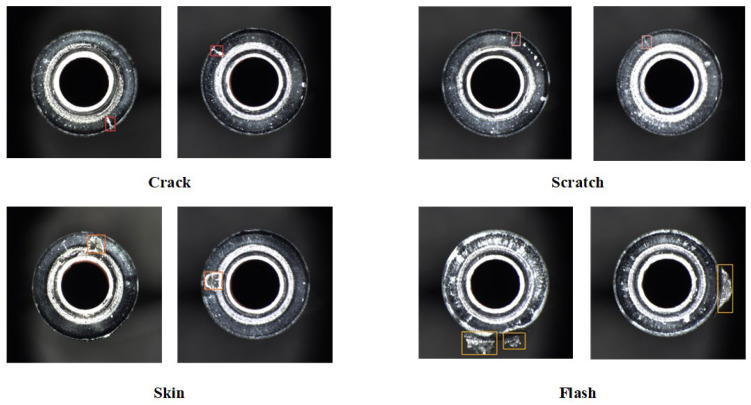
Types of surface defects in brake pipe ends.

The brake pipe (see Figure 7) end surface defect dataset used in this study was captured by an industrial camera in a real-world environment, ensuring high-quality image acquisition. Based on different defect attributes, the defects are classified into four types: cracks, scratches, flash, and skin, as depicted in Figure 8. The Python-based annotation tool Labelimg was used to label the collected defect images with rectangular bounding boxes along with their corresponding categories. This dataset encompasses the main defect types that may occur during the ends’ production phase, and consists of 1291 images. The distribution of defects among each type is presented in Table 1. The dataset was divided into training, validation, and test sets in a ratio of 7:1.5:1.5. Subsequently, the input image size was resized to 640 × 640 pixels.

### 4.2. Evaluation Metrics

The evaluation metrics in this paper encompass both performance and complexity dimensions. Regarding performance, we adopt Precision (P), Recall (R), Average Precision (AP), mean Average Precision (mAP), and the F1-score, which all commonly applied in object detection. Equations (10)–(13) present the formulas for these metrics. In these equations, TP represents true positives (i.e., correctly classified positives), FP represents false positives (i.e., incorrectly classified positives), and FN represents false negatives (i.e., positives incorrectly classified as negatives). Here, k represents the number of defect types, which is set to 4 in this paper. Additionally, mAP50 evaluates the average precision of predicted bounding boxes with an IoU threshold higher than 0.5. The F1-score, calculated as the harmonic mean of Precision and Recall, offers a comprehensive and balanced assessment of the model’s overall performance.

(10) $$P=\frac{T_{p}}{T_{p}+F_{p}}$$

(11) $$R=\frac{T_{p}}{T_{p}+F_{N}}$$

(12) $$mAP=\frac{\sum_{i=1}^{k}AP_{i}}{k}$$

(13) $$F_{1}=2\times \frac{P\times R}{P+R}$$

This study examines model complexity from the perspectives of parameter count, frame per second (FPS), and floating point operations (FLOPs). FLOPs, or Floating Point Operations Per Second, are an important statistic for evaluating the computational burden of neural network models. Lower FLOPs values signify less computing complexity [39].

### 4.3. Environment and Training Parameter Settings

The software environment employed for this experiment comprises Windows 10, Python 3.8, CUDA 11.8, and PyTorch 2.2.2. The hardware configuration encompasses an Intel(R) Xeon(R) W-2145 CPU with a clock speed of 3.70 GHz, 64 GB of RAM, and an NVIDIA GeForce RTX 2080 Ti GPU with 12 GB of memory. The crucial parameters for network training are summarized in Table 2.

The learning rate is a critical hyperparameter influencing model convergence. Higher learning rates expedite weight updates and can accelerate convergence, but risk instability or hinder training if they are excessively high. For YOLOv8, an initial range of 0.1 to 0.00001 was explored, with a learning rate of 0.001 identified as optimal based on experimental results.

Weight decay, serving as a regularization factor in the loss function, mitigates overfitting and enhances generalization. For YOLOv8, a value of 0.937 was found to balance generalization and stability, aligning with its recognized importance in complex models.

Batch size, another pivotal hyperparameter, was set to 32 to optimize computational efficiency and maintain stable gradient updates. Although slightly less accurate than a batch size of 16, this configuration significantly improved training speed and resource utilization. On the end-head dataset, a batch size of 16 achieved an mAP50 of 86.6%, while a batch size of 32 resulted in 86.2%, demonstrating minimal accuracy loss for substantial efficiency gains.

### 4.4. Ablation Experiment

To evaluate the effectiveness of the proposed FP-YOLOv8 model for end surface defect detection, a series of ablation experiments were conducted using YOLOv8n as the baseline. The results are summarized in Table 3. The modules A, B, and C in the table represent the C2f_GhostV2, Dynamic ATSS, and FADH modules, respectively, with √ next to A, B, and C indicating that these modules were added to the baseline model. Experimental results demonstrate that integrating the C2f_GhostV2 module reduces the number of parameters and computations by 14.3% and 21.0%, respectively, although it slightly lowers mAP50 and F1-score. This module leverages multiple Ghost modules to efficiently generate feature maps with fewer parameters through point-wise and depth-wise convolutions, thereby reducing the model’s complexity. Despite this, the layer count increases from 185 to 257, enhancing the model’s capacity for hierarchical feature learning and improving representational ability. Replacing the global attention mechanism with the DFC attention mechanism further reduces computational cost while strengthening feature representation. Upon introducing Dynamic ATSS, mAP50, and F1-score improved by 2.3% and 1.0%, respectively. This improvement is attributed to the adaptive label assignment strategy, which dynamically adjusts the IoU threshold, correctly classifying more high-quality samples as positive and enhancing detection accuracy. Following the integration of FADH, the model achieved an accuracy of 83.2%, recall of 86.2%, mAP50 of 89.0%, and an F1-score of 84.0%, improving by 2.9%, 4.0%, 2.8%, and 3.0%, respectively. These results demonstrate that FADH effectively enhances small-target detection by leveraging depth-wise separable convolutions to optimize feature extraction and reduce computational costs. The decoupled design minimizes task coupling, allowing for more precise learning of classification and regression tasks. This balance between efficiency and accuracy underscores FADH’s suitability for improving small-target defect detection.

Furthermore, this paper investigates the performance of different combinations of modules A, B, and C. Integrating module B into module A improves mAP50 by 1.6%, reaching 86.2%, and raises the F1-score to 81%, while reducing the parameter count and computational cost by 14.3% and 21.0%, respectively. Ghost Modules within C2f_GhostV2 efficiently generate additional feature maps through simple linear transformations. To balance efficiency and effectiveness, the DFC attention mechanism operates on down-sampled features (halved in width and height) to cut 75% of its FLOPs, with up-sampling applied afterward to restore resolution. This combination, alongside Dynamic ATSS, which dynamically refines label allocation, results in improved accuracy and adaptability to complex scenes.

For the B + C combination, the model achieves a mAP50 of 84.6% and an F1-score of 83%. FADH, with its depth-wise separable convolutions, strengthens small-object detection, while its decoupled detection head optimizes classification and regression tasks. Dynamic ATSS refines sample assignment, enabling the model to better handle the challenges posed by diverse object shapes and sizes.

The combination of A + C yields significant improvements. mAP50 increases by 2.8%, reaching 89%, and detection accuracy improves to 87.6%, reflecting a 7.3% gain compared to the baseline model. Remarkably, these improvements are achieved while maintaining the same parameter count and computational cost as the A model. The DFC attention mechanism of C2f_GhostV2 captures precise spatial relationships, laying a foundation for feature enhancement. Simultaneously, the depth-wise convolutions in FADH fully utilize these enhanced features, achieving fine-grained feature representation and improved small-object detection. The synergy between C2f_GhostV2 and FADH demonstrates their strong complementarity, elevating the performance of the A + C model to new heights.

When all three modules are integrated into the FP-YOLOv8 model, significant improvements are observed. Precision, recall, mAP50, and F1-score increase by 4.3%, 8.6%, 3.3%, and 6.0%, respectively, compared to the baseline. Both computational cost and parameter count are reduced by 21.0% and 14.3%, respectively. This synergy between C2f_GhostV2, FADH, and Dynamic ATSS enhances feature representation and model efficiency, leading to substantial performance gains. C2f_GhostV2 focuses on reducing redundant features and capturing long-range spatial dependencies, while FADH strengthens fine-grained feature extraction and small-object detection. Dynamic ATSS improves detection accuracy by dynamically adjusting label assignments.

In this paper, each defect type was analyzed independently to comprehensively investigate the impact of different modules on the detection of diverse defect types. The results are presented in Table 4, where Cr, Sc, Sk, and Fl stand for cracks, scratches, skins, and flash defects, respectively, and modules A, B, and C represent the C2f_GhostV2, Dynamic ATSS, and FADH modules, respectively, with √ next to A, B, and C indicating that these modules were added to the baseline model.

The integration of the C2f_GhostV2 module resulted in a reduction in model parameters and computational complexity, accompanied by a modest decrease in detection accuracy for certain defect types. Specifically, the accuracy for cracks, scratches, and flash defects decreased by 0.4%, 1.5%, and 7.5%, respectively, while skin defect detection improved by 3.2%. These results highlight the efficiency gains achieved by C2f_GhostV2, although they also indicate a trade-off in precision, particularly for flash defects, while suggesting potential benefits for skin defect detection.

For the Dynamic ATSS module, upon introduction, the detection accuracies for cracks, scratches, skins, and flash defects improved by 4.3%, 3.2%, 0.9%, and 0.8%, respectively, relative to the baseline. This clearly shows that the adaptive label assignment within Dynamic ATSS significantly enhances the detection accuracy across all defect types. Notably, it is especially beneficial for small targets in complex backgrounds, highlighting its crucial role in improving the detection of such challenging scenarios within different defect types.

With the introduction of FADH module, the detection accuracies for cracks, scratches, and skins improved by 6.8%, 0.3%, and 4.5%, respectively, although the detection accuracy for flash defects slightly decreased. This indicates that FADH is more effective in capturing detailed information at different scales, thereby improving the detection of small-object defects like cracks, scratches, and skins. It demonstrates that FADH has a distinct advantage over the original detection head of YOLOv8 in handling specific small-scale features within these defect types.

When C2f_GhostV2, Dynamic ATSS, and FADH are combined, the detection accuracies for cracks, scratches, skins, and flash defects reach 81.8%, 95.4%, 91.8%, and 89.1%, respectively. Compared to the baseline, there are improvements of 5.5%, 5.6%, and 2.3% in the detection of cracks, scratches, and flash defects. This combined model demonstrates excellent performance with enhanced sensitivity towards small-target defects. It implies that the synergy among these modules effectively addresses the challenges in detecting different defect types, particularly those related to small targets.

**Figure 9:**
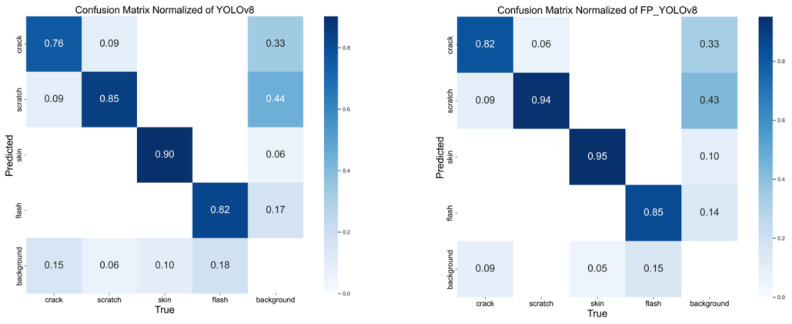
Confusion matrix.

Additionally, the confusion matrices of the baseline YOLOv8n model and the FP-YOLOv8 model are presented in Figure 9. The analysis of these matrices reveals that FP-YOLOv8 outperforms YOLOv8n by achieving a higher proportion of correctly classified defect types and a lower proportion of misclassifications of defect types as background. This further demonstrating the superior detection capability of FP-YOLOv8 in handling various defect types, providing more reliable results in the defect detection process.

**Figure 10:**
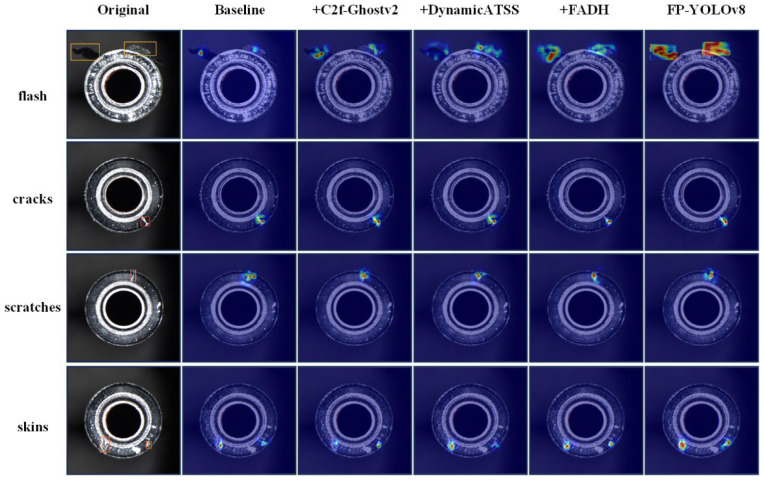
Comparison of heatmaps for different algorithms across four types of defects.

To visually emphasize the performance gains of the proposed algorithm, heat maps generated using HiResCAM for defect identification on the end surface under the influence of different modules are shown in Figure 10. It is evident from the figure that FP-YOLOv8 effectively focuses on defect locations and emphasizes the features of target defects in the foreground, with minimal interference from background noise.

### 4.5. Comparative Experiments on the Ends Surface Defect Dataset

To demonstrate the effectiveness of the proposed FP-YOLOv8 algorithm, we conducted a series of experiments using the ends surface defect dataset and compared it with several established target detection models. The results presented in Table 5 indicate that models such as YOLOv8n, Faster-RCNN, SSD, and Retina-Net have inferior detection accuracy, accompanied by significantly higher parameter counts and computational requirements, making them challenging to deploy on resource-limited devices. Compared to other YOLO models, YOLOv3-Tiny [40] and YOLOv6n [41] fall behind YOLOv8n in both detection accuracy and parameter efficiency. While YOLOv5n [42] has a simplified structure, it does not show significant improvements in detection. Although YOLOv10n [43] is a newer model, it does not outperform YOLOv8n. Based on a comprehensive analysis of detection accuracy, model parameters, and computational cost, YOLOv8n serves as the baseline model in this paper.

**Figure 11:**
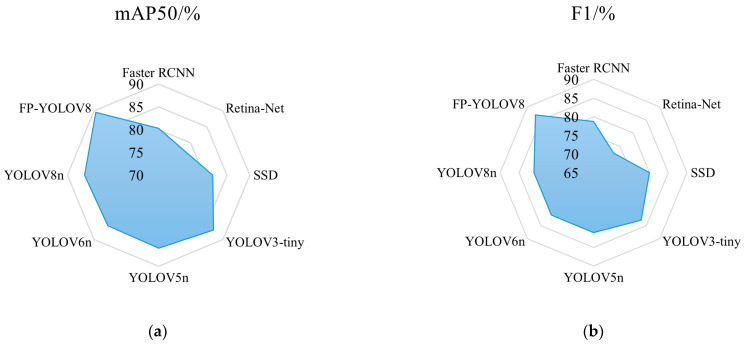
Score values of each model; (**a**) mAP50; (**b**) F1-score.

**Figure 12:**
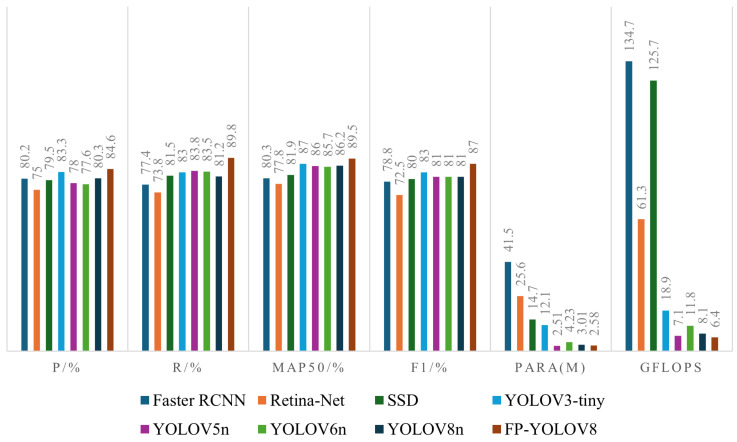
Comparison of each parameter of each model.

As indicated in Table 5, the FP-YOLOv8 model surpasses other models in mAP50, F1-score, and computational efficiency, providing the most effective balance between detection accuracy and model complexity. Specifically, compared with the baseline YOLOv8n, FP-YOLOv8 improves mAP50 and F1-score by 3.3% and 6.0%, respectively, while reducing the number of parameters by 14.3% and the GFLOPs by 21.0% (from 8.1 to 6.4). Additionally, FP-YOLOv8 achieves a higher detection speed (FPS) compared to YOLOv8n. Compared with the two-stage detector Faster-RCNN, FP-YOLOv8 has only 6.2% of the parameters and requires just 4.8% of the computation, with a reduction of 128.3M GFLOPs. To further illustrate the performance differences, the metrics in Table 5 are presented in both radar charts (Figure 11) and bar charts (Figure 12). As shown in Figure 11, FP-YOLOv8 achieves the best detection performance for ends surface defects. Figure 12 shows that FP-YOLOv8 delivers higher detection accuracy while consuming fewer computational resources.

Table 6 presents the detection accuracies of different models for four types of defects: cracks, scratches, skins, and flash defects. The results indicate that FP-YOLOv8 significantly outperforms other models in detecting cracks, scratches, and flash defects. While its performance in detecting skin defects is slightly lower than that of YOLOv3-Tiny, FP-YOLOv8 still achieves competitive accuracy. Overall, FP-YOLOv8 strikes an excellent balance between detection accuracy, model size, and computational complexity. It offers the advantages of a compact model, reduced computation, and fast detection, making it highly suitable for practical applications.

### 4.6. Comparative Experiments on the NEU-DET Dataset

**Figure 13:**
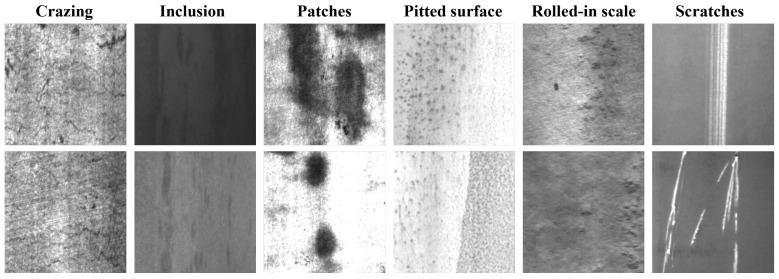
Illustration of six types of defects in the NEU-DET dataset.

The detailed experimental analysis in the previous section firmly proves the proficiency of the FP-YOLOv8 model on the end surface defect dataset. To further explore its generalization ability and evaluate its effectiveness in identifying minute targets amidst intricate backgrounds, we conducted a further evaluation of the model using the NEU-DET dataset [44] from Northeastern University. This dataset encompasses 1800 grayscale images, illustrating six common surface defects in hot-rolled strip steel, including post-roll oxide skin (RS), spots (Pa), silvering (Cr), pockmarks (PS), inclusions (In), and scratches (Sc), as depicted in Figure 13. The images were divided into training and validation sets at a ratio of 7:3, and a larger test set was used to assess the model’s performance on unseen data, thereby providing a thorough evaluation of its generalization ability. The experimental training parameters were set in accordance with the guidelines presented in Section 4.3 of this paper.

As shown in Table 7, although FP-YOLOv8 has a slightly larger number of parameters than YOLOv5n, it outperforms both YOLOv5n and YOLOv8n in terms of detection accuracy and computational efficiency. Specifically, FP-YOLOv8 shows a 3.2% improvement in detection accuracy over YOLOv5n and a 2.9% improvement over YOLOv8n. Furthermore, FP-YOLOv8 outperforms YOLOv10 and YOLOv11 [45] by 3.7% and 1.3% in detection accuracy, respectively, while maintaining similar parameter counts and computational complexity. These results demonstrate significant improvements in detection performance on the ends surface defect dataset and substantial enhancements on the NEU-DET steel surface defect dataset, validating the model’s effectiveness and strong generalization capability. The experiment further confirms the proposed method’s ability to enhance detection performance.

### 4.7. Hyperparameter Sensitivity Analysis Experiment

**Figure 14:**
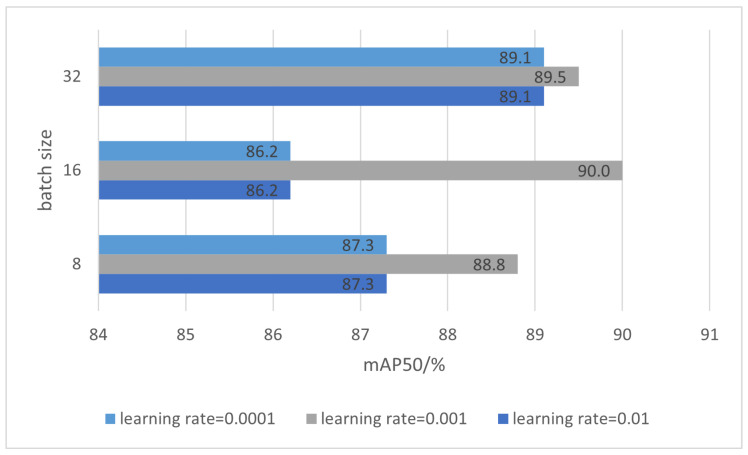
Experiments on sensitivity analysis of learning rate and batch size hyperparameters.

To analyze the sensitivity of the model to hyperparameters, we conducted experiments by varying only the learning rate and batch size, while keeping all other hyperparameters fixed, as described in Section 4.3. The experimental results demonstrate that the learning rate has a significant impact on model performance, with a learning rate of 0.001 consistently achieving the highest accuracy across all batch sizes. Specifically, the combination of a 0.001 learning rate and a batch size of 16 provides the best performance, reaching an accuracy of 90, as shown in Figure 14. This indicates that the model converges most effectively under this configuration.

In contrast, learning rates of 0.01 and 0.0001 yield slightly lower and comparable accuracy values, suggesting that these settings may either cause the model to converge too quickly (in the case of 0.01) or too slowly (in the case of 0.0001). These results highlight the sensitivity of the model to learning rate adjustments.

Regarding batch size, a larger batch size, such as 32, tends to provide better performance for suboptimal learning rates (0.01 and 0.0001), likely due to improved stability in gradient estimation. However, for the optimal learning rate of 0.001, a batch size of 16 outperforms both smaller (8) and larger (32) batch sizes, indicating a balanced trade-off between gradient noise and computational efficiency.

In summary, the results suggest that careful tuning of both learning rate and batch size is critical for achieving optimal model performance.

### 4.8. Practical Application

**Figure 15:**
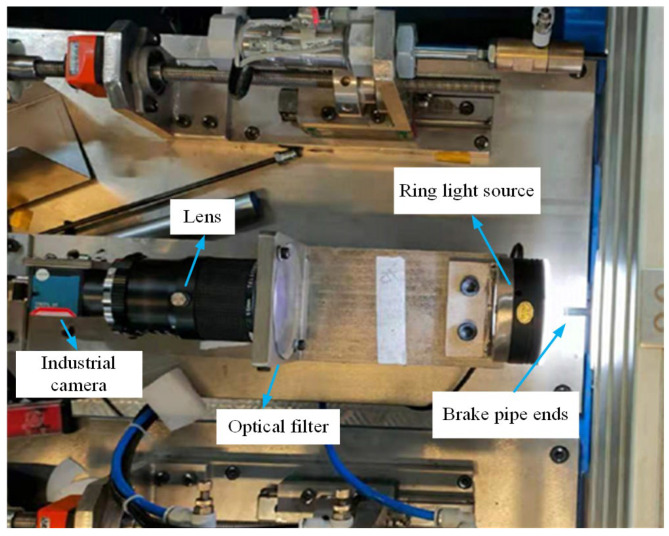
In-line visual inspection device for brake pipe ends.

**Figure 16:**
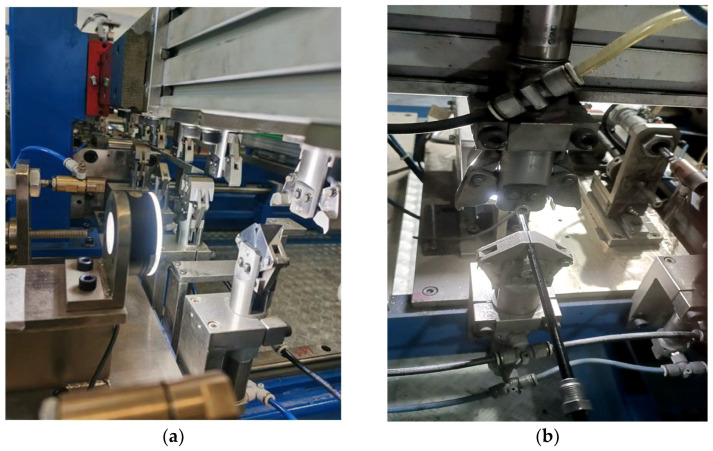
Actual production environment application. (**a**) shows the light source and clamp in actual manufacturing, (**b**) shows the automotive brake pipe being transferred from the clamp to the vision inspection system for defect detection.

**Figure 17:**
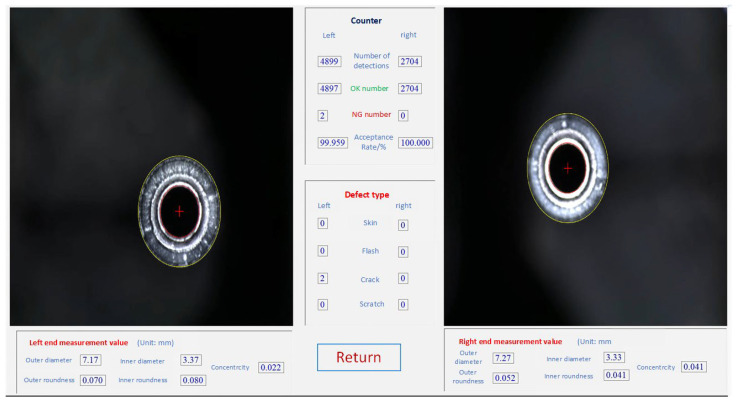
Brake pipe ends measurement result display interface. The yellow circle represents the outer circle of the brake pipe end and the red circle represents the inner circle of the brake pipe end.

The improved FP-YOLOv8 algorithm has been successfully implemented in practical production situations, displaying higher accuracy, processing speed, overall robustness, and even greater lightness in terms of computational burden and resource usage. Figure 15 illustrates the online image acquisition device used in the production setting. As illustrated in Figure 16, the brake pipe is transferred to the visual inspection station via a clamp mechanism. The inspection system detects defects and sorts the brake pipes based on the presence or absence of defects. Figure 17 presents the detailed inspection result interface, which provides real-time displays of the enlarged image of the inspected brake pipe ends, along with various dimensional parameters and defect statistics.

## 5. Conclusions

The FP-YOLOv8 algorithm presented in this paper effectively addresses the challenges of detecting surface defects on brake pipe ends by optimizing both accuracy and computational efficiency. Through the integration of three novel modules—C2f_GhostV2, Dynamic ATSS, and FADH—this model achieves superior performance compared to the baseline YOLOv8n model.

The C2f_GhostV2 module reduces model parameters by 14.3% and computational load by 21.0%, ensuring a lightweight structure without significantly sacrificing accuracy. Dynamic ATSS improves label assignment precision by dynamically adjusting IoU thresholds, which contributes to a 2.3% increase in mAP50 and a 1.0% boost in F1-score, especially enhancing accuracy for small targets. The FADH module further increases detection precision for small defects, such as cracks and scratches, by capturing finer details at multiple scales. Collectively, these innovations result in an overall mAP50 of 89.5% and an F1-score of 87.0%, representing respective improvements of 3.3% and 6.0% over YOLOv8n.

Additionally, the model achieves notable gains in detecting specific defect types, with AP50 values improving by 5.5% for cracks, 5.6% for scratches, and 2.3% for flash defects. Validation on the NEU-DET dataset further demonstrates the model’s strong generalization ability, with a 2.9% improvement in mAP50 over YOLOv8n, a 3.7% improvement over YOLOv10n and a 1.3% improvement over YOLOv11n. These results confirm the model’s potential for online application in manufacturing environments, fulfilling the requirements for high accuracy and high efficiency that are essential for effective defect detection on the surfaces of brake pipe ends. Despite the significant improvements achieved by FP-YOLOv8, several limitations persist. Both the NEU-DET and brake pipe end datasets feature relatively simple backgrounds and similar defect types. However, the model’s performance may be adversely affected in more complex real-world scenarios. Specifically, in environments with intricate backgrounds, occlusion, or variable lighting conditions, detection accuracy is likely to decline due to these additional complexities. Furthermore, the model’s reliance on high-quality labeled data for training remains a significant limitation. The availability of such labeled data is often scarce, particularly in industrial settings, making it challenging to generalize the model to new datasets or previously unseen defect categories without extensive retraining.

To address these challenges, future work will focus on two key directions. First, advanced image preprocessing techniques and customized data augmentation strategies will be developed to enhance the model’s robustness against complex backgrounds, occlusion, and lighting variations. Structural optimizations will also be explored to improve detection accuracy for challenging defects, such as cracks, while maintaining the model’s lightweight design, so that it can be easily deployed on devices with limited resources in the future. Second, unsupervised and semi-supervised learning approaches will be investigated to reduce the model’s reliance on large annotated datasets, thus improving its adaptability to new industrial environments. These advancements will contribute to making FP-YOLOv8 more effective and efficient for real-world defect detection in complex, dynamic industrial conditions.

## Figures and Tables

**Table 1:** The number of images of the defect type in the dataset.

Defect Type	Cracks	Scratches	Flash	Skin
Number of images	316	331	262	382

**Table 2:** Experimental parameter settings.

Parameter	Value
Initial learning rate	0.001
Final learning rate	0.0001
Momentum	0.937
Weight decay	0.0005
Epoch	300
Batch size	32

**Table 3:** Analysis of detection effects of algorithms using different modules.

A	B	C	P/%	R/%	mAP50/%	F1/%	Para/M	GFLOPs	Layers
Baseline			80.3	81.2	86.2	81.0	3.01	8.1	185
√			77.5	81.8	84.6	80.0	2.54	6.8	257
	√		78.6	86.1	88.5	82.0	3.01	8.1	185
		√	83.2	86.2	89.0	84.0	3.05	7.7	194
√	√		79.0	83.6	86.2	81.0	2.54	6.8	257
	√	√	83.0	83.9	84.6	83.0	3.05	7.7	194
√		√	87.6	84.0	89.0	86.0	2.58	6.4	266
√	√	√	84.6	89.8	89.5	87.0	2.58	6.4	266

**Table 4:** The detection AP50 corresponds to the detection result of the four defects by each algorithm.

A	B	C	AP 50 (%)			
			Cr	Sc	Sk	Fl
Baseline			76.3	89.8	91.8	86.8
√			79.5	89.4	90.3	79.3
	√		80.6	93.0	92.7	87.6
		√	83.1	90.1	96.3	86.5
√	√	√	81.8	95.4	91.8	89.1

**Table 5:** The comparison of detection accuracy of different models on the brake pipe end surface defect dataset.

Model	Backbone	P/%	R/%	mAP50/%	F1/%	Para (M)	GFLOPs	FPS
Faster-RCNN	ResNet-50	80.2	77.4	80.3	78.8	41.5	134.7	
Retina-Net	ResNet-50	75.0	73.8	77.8	72.5	25.6	61.3	
SSD	Vgg16	79.5	81.5	81.9	80.0	14.7	125.7	
YOLOV3-tiny		83.3	83.0	87.0	83.0	8.69	12.9	74.8
YOLOV5n		78.0	83.8	86.0	81.0	2.51	7.1	63.5
YOLOV6n		77.6	83.5	85.7	81.0	4.23	11.8	74.0
YOLOV8n		80.3	81.2	86.2	81.0	3.01	8.1	64.3
YOLOV10n		82.1	80.9	84.8	81.0	2.58	7.8	62.2
FP-YOLOV8		84.6	89.8	89.5	87.0	2.58	6.4	65.7

**Table 6:** The detection AP50 corresponding to the detection results of the four defects using each model.

Model	Backbone	AP 50 (%)			
		Cr	Sc	Sk	Fl
Faster-RCNN	ResNet-50	69.9	81.3	89.3	80.8
Retina-Net	ResNet-50	64.0	84.0	89.0	74.0
SSD	Vgg16	72.5	84.9	86.5	83.8
YOLOv3-tiny		79.4	90.2	95.6	82.7
YOLOv5n		75.6	90.7	93.6	84.8
YOLOv6n		74.4	89.2	93.2	86
YOLOv8n		76.3	89.8	91.8	86.8
YOLOv10n		76.4	91.5	92.2	79.0
FP-YOLOv8		81.8	95.4	91.8	89.1

**Table 7:** Detection results of NEU-DET dataset.

Model	mAP50/%	Para (M)	GFLOPs
SSD	67.3	14.7	124.9
Retina-Net	62.4	25.6	61.3
YOLOv5n	74.5	2.51	7.1
YOLOv8n	74.8	3.01	8.1
YOLOv10n	74.0	2.58	7.8
YOLOv11n	76.4	2.58	6.3
FP-YOLOv8	77.7	2.58	6.4

## Data Availability

The brake pipe ends dataset presented in this study are available upon request from the corresponding author, as the dataset is used for actual company production. The NEU-DET dataset is available at https://github.com/raoke0/NEU-DETdataset.git (accessed on 21 November 2024).
